# Airborne Bacterial Communities in Residences: Similarities and Differences with Fungi

**DOI:** 10.1371/journal.pone.0091283

**Published:** 2014-03-06

**Authors:** Rachel I. Adams, Marzia Miletto, Steven E. Lindow, John W. Taylor, Thomas D. Bruns

**Affiliations:** Department of Plant & Microbial Biology, University of California, Berkeley, California, United States of America; Field Museum of Natural History, United States of America

## Abstract

Genetic analysis of indoor air has uncovered a rich microbial presence, but rarely have both the bacterial and fungal components been examined in the same samples. Here we present a study that examined the bacterial component of passively settled microbes from both indoor and outdoor air over a discrete time period and for which the fungal component has already been reported. Dust was allowed to passively settle in five common locations around a home − living room, bedroom, bathroom, kitchen, and balcony − at different dwellings within a university-housing complex for a one-month period at two time points, once in summer and again in winter. We amplified the bacterial 16S rRNA gene in these samples and analyzed them with high-throughput sequencing. Like fungal OTU-richness, bacterial OTU-richness was higher outdoors then indoors and was invariant across different indoor room types. While fungal composition was structured largely by season and residential unit, bacterial composition varied by residential unit and room type. Bacteria from putative outdoor sources, such as *Sphingomonas* and *Deinococcus*, comprised a large percentage of the balcony samples, while human-associated taxa comprised a large percentage of the indoor samples. Abundant outdoor bacterial taxa were also observed indoors, but the reverse was not true; this is unlike fungi, in which the taxa abundant indoors were also well-represented outdoors. Moreover, there was a partial association of bacterial composition and geographic distance, such that samples separated by even a few hundred meters tended have greater compositional differences than samples closer together in space, a pattern also observed for fungi. These data show that while the outdoor source for indoor bacteria and fungi varies in both space and time, humans provide a strong and homogenizing effect on indoor bacterial bioaerosols, a pattern not observed in fungi.

## Introduction

The bioaerosol component of the built environment is a well-defined territory that lends itself to the study of microbial dispersal, by allowing one to ask basic questions about sources and processes that define these aerosols. The source populations for these indoor, airborne microbes are either the outdoors or an indoor surface with subsequence aerosolization. The processes that have been shown to structure indoor environments include geography and climate [1], seasons [2], building design and ventilation system [3], [4], and the presence of pets along with human inhabitants and their behavioral patterns [5], [6].

Studies to date on the microbiology of the built environment suggest that different processes structure bacteria and fungi. Studies targeting bacteria show a marked signal of human-associated taxa and implicate humans as an agent of dispersal for soil-associated taxa [6]–[9]. For example Dunn et al [6] found that in homes, the bacteria associated with the oral cavity are often found on pillowcases and those with human skin and stool on the toilet set, while the bacterial communities on the handle of toilets in public restrooms can be similar to those on the floor [9]. Fungi, on the other hand, show little direct influence of humans and exhibit geographic structure on a global [1] and even a local scale [10]. In contrast to bacteria, the focus on fungi has traditionally been on detecting surface growth especially in water-damaged buildings (e.g., [11]).

Here we report results sampling bacterial communities in residences for which we have already reported on the fungal component [10]. The study design implemented here has several notable features. One is a paired outdoor sample, in addition to replicated indoor samples both within and across residential units. When outdoor samples are included, bacterial studies haven shown a strong influence of the dynamic outdoor source, in addition to other factors on indoor air [3], [4]. By collecting airborne microbes that passively settled on a sampling device, we have samples originating from a discrete yet long-term time period. Plus, replicated dwellings of similar design were simultaneously sampled, supporting statistical analyses that identify individual processes structuring these indoor environments. Time-resolved data on both bacterial and fungal diversity in the same outdoor and indoor samples allowed us to show that processes structuring their indoor communities have important differences as well as commonalities.

## Materials and Methods

The study location and collection methods were described previously [10]. Briefly, residences were distinct, family units of a university-housing complex that were uniform in floor plan, building material, resident turnover, mechanical ventilation, and the absence of pets. Bioaerosols were passively collected on suspended, open-faced, empty plastic petri dishes that were suspended from the ceiling at a height of approximately 2.5 m and placed at least one meter from a vent (see Figure S2 of [10]). Samplers were exposed for a period of one month. In each of the dwellings four samplers were located indoors in the kitchen, living, bathroom, and bedroom, and one sampler was placed outdoors on the balcony. The experiment surveyed 11 units in the summer and eight of those same units the following winter.

At the time the experiment was started, residents answered questions on unit floor plan, inhabitants, and their behavior that were subsequently tested as possible explanatory factors: number of bedrooms, bathrooms, and residents; presence of houseplant(s), and use of humidifier. Four self-reported survey topics were invariant and therefore excluded: use of air treatment, typical occupancy during the day, and frequency of cleaning and opening of windows. The sampling protocol was conducted under approval by the University of California’s Committee for the Protection of Human Subjects, Protocol ID #2011-03-2947, and approved by both the Village Residents Association for the housing complex (May 18, 2011) and the Residential and Student Service Programs of the University (July 25, 2011).

### Molecular Analysis

Settled microbes and dust were collected from the dish surface using a moistened sterile cotton swab. Cell lysis and nucleic acid isolation from the swab tip relied on an initial bead-beating in phenol:chloroform, followed by treatment with the MoBio Power Soil DNA extraction kit (Carlsbad, CA, USA) (Protocol S1). Of the 95 samples originally collected, 59 retained sufficient extraction volume to be used for pyrosequencing of PCR amplified bacterial DNA (Table S1). Chi-square analysis revealed that the 36 samples excluded due to low extraction volume did not differ significantly from the 59 included samples based on season, indoor/outdoor category, or room type. A 300 bp region targeting the V1/V2 region of the 16S rRNA gene was amplified in triplicate and pooled using primers 8f/357r modified for 454 pyrosequencing (Protocol S1). The pooled amplified products were cleaned with AMPure magnetic beads (Beckman Coulter Genomics, Danvers, MA). Amplicon concentration was determined using the Qubit fluorometer (Invitrogen, Carlsbad, CA, USA), and combined at a 25 ng equimolar concentration for downstream sequencing. Samples were split across three different runs at the University of Illinois. All raw sequences, including those samples with low amplification yield that were excluded from this analysis, have been deposited into NCBI’s SRA with accession SRP030126 (Table S1).

The relative bacterial biomass of the original samples was estimated from the Qubit measurement of the concentration of PCR-amplified DNA, a practice supported by our previous work showing a strong overlap between the relative Qubit-determined concentration of PCR-amplicons and spore-equivalent biomass as determined in quantitative PCR (unpublished, Figure S1).

### Data Analysis

Sequence analysis relied on the software UPARSE [12], QIIME [13], and R [14]. Using scripts on drive5 [15] related to UPARSE, the fasta and qual files of each of the 454 runs were converted to a.fastq file, and these three files were then concatenated into a single.fastq file for analysis in the UPARSE pipeline [16]. Sequences were filtered to a fixed length of 150 base pairs (as variable lengths can lead to errors during dereplication of reads: [12]) and those with an expected error probability greater than 0.5 were discarded. Singleton reads were excluded before clustering. Chimeras were checked against the “Gold” database based on UCHIME [17], [18], and reads were clustered at 97% sequence identity into operational taxonomic units (OTUs). This UPARSE pipeline was used to assign final OTUs and create the community table detailing samples and the OTUs present in each sample. QIIME was then used to align the OTU sequences based on MUSCLE [19], construct the phylogenetic tree, compute distance matrices between samples, and assign taxonomy using the rdp classifier method against the Greengenes database [20], version updated May, 2013. Differences in community composition were determined by both the taxon-based, binary Bray-Curtis distance and the phylogenetically-informed weighted Unifrac distance [21] implemented in QIIME.

OTUs in negative controls (n = 9; Table S2) were excluded from all samples. Thirty-two phylotypes were classified as chloroplasts and removed from the table. The dominant chloroplast types were from *Pinus sp.* and *Quercus sp.*, common tree species in the area, and a common, local weed, *Medicago sp*. Excluding chloroplasts in some cases greatly reduced the number of sequence reads, particularly in outdoor winter samples which then were replicated in only one instance. In total, 50 samples could be analyzed, 39 indoor and 11 outdoor (Table S1).

Ecological analysis and visualization of results relied on R [14]. To achieve an even sampling depth per extracted sample, the dataset was rarefied to 100 sequence reads per sample which has been shown to be sufficient for identifying differences in microbial communities [22]. To look for broad differences in OTU richness across different sampling groups – such as seasons, units, and locations within a unit – we used the Kruskal-Wallis test when there were three or more groups and the Mann-Whitney test for two groups. Compositional differences were depicted using visualization of principal coordinates analysis (PCO). Statistical predictors of community composition were analyzed using PERMANOVA implemented by ADONIS [23] based on both binary Bray-Curtis and Unifrac community distances. Taxa indicative of potential source environments (i.e. source tracking) for bacteria were identified from other studies in which they found taxa highly associated with a particular environment [6], [9]. For example, the family *Corynebacteriaceae* was consistently associated with human skin. The mean relative abundances of the different indicator taxa in different sample types were represented as a heatmap. Correlations between distance matrices for taxon composition and geographic location were analyzed using the Mantel test.

In order to directly compare the bacterial and fungal data sets, we reanalyzed the fungal amplicon reads [10] using the same bioinformatic UPARSE pipeline detailed above, with the exception that sequences were filtered to a fixed length of 100 base pairs (as the ITS1 region of some fungal lineages can be this length) and chimeras were checked against the UNITE database [24]. Community composition between fungi and bacteria is compared using the Bray-Curtis index, since the phylogenetically-informed Unifrac metric is not appropriate for the ITS marker.

## Results

### OTU Richness

**Figure 1:**
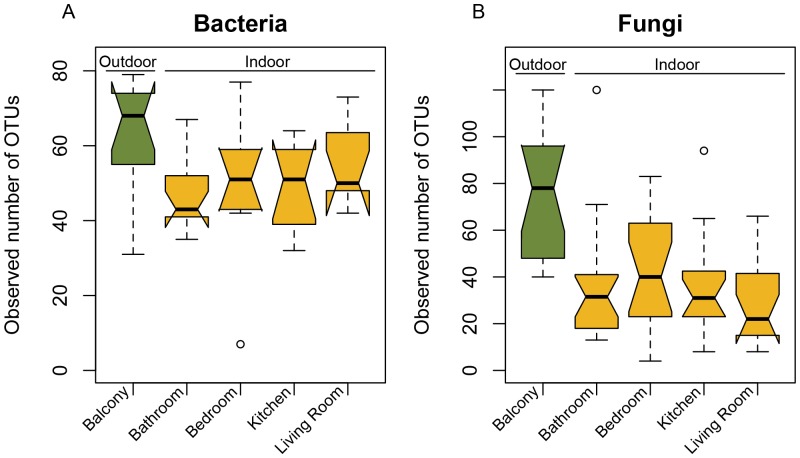
Bacterial richness (A) and fungal richness (B) across sample locations. Both bacterial and fungal OTU richness was higher outdoors than indoors. Solid lines represent the median, boxes the quartiles, and bars the interquartile range. Outliers are circles.

We detected 849 bacterial taxa across our samples, 770 found indoors (sample number = 39) and 557 outdoors (sample number = 11). Observed richness was higher outdoors than indoors (Mann-Whitney test, p = 0.02; Figure 1A) but richness on the balcony was not significantly higher than any of the indoor rooms (pairwise Mann-Whitney tests, p>0.05). Observed bacterial richness was also not different across indoor rooms (Kruskal-Wallis test, p>0.05). Bacterial richness tended to be higher in those four (of 11) units that reported at least occasional humidifier use (Mann-Whitney test, p = 0.07; mean in group yes = 52.7; mean in group no = 48.0). No other measured factors were correlated with observed differences in bacterial richness, including season. Richness comparisons based on the Shannon diversity metric were identical to observed richness.

Similar to these observed bacterial richness patterns, observed fungal richness was higher outdoors than indoors (Mann-Whitney test, p<0.01; Figure 1B) and was invariant across indoor rooms (Kruskal-Wallis test, p>0.05). Unlike bacteria, fungal richness on the balcony was significantly higher than each of the indoor rooms (pairwise Mann-Whitney test, p<0.02).

### Community Composition

The most common OTUs was classified as *Sphingomonas* sp., representing 3.0% of all sequences, and this bacterium was more abundant outdoors (5.9% of sequences) than indoors (1.6%). Conversely, the next two most common bacterial OTUs were both *Staphylococcus* spp. and they were much more common indoors (4.2% and 4.0%) than outdoors (0.02% and 0.02%). Generally, those taxa abundant outdoors were also present indoors, while the reverse was not true. Only one of the 50 most abundant outdoor taxa was not observed indoors – *Deinococcus aquatilis*. On the other hand, 29 of the 50 most abundant indoor taxa were not observed outdoors or were represented in three or fewer sequence reads. This contrasts with abundance patterns in fungi, in which all abundant taxa indoors were also abundant outdoors (Table 2 in [10]).

**Figure 2:**
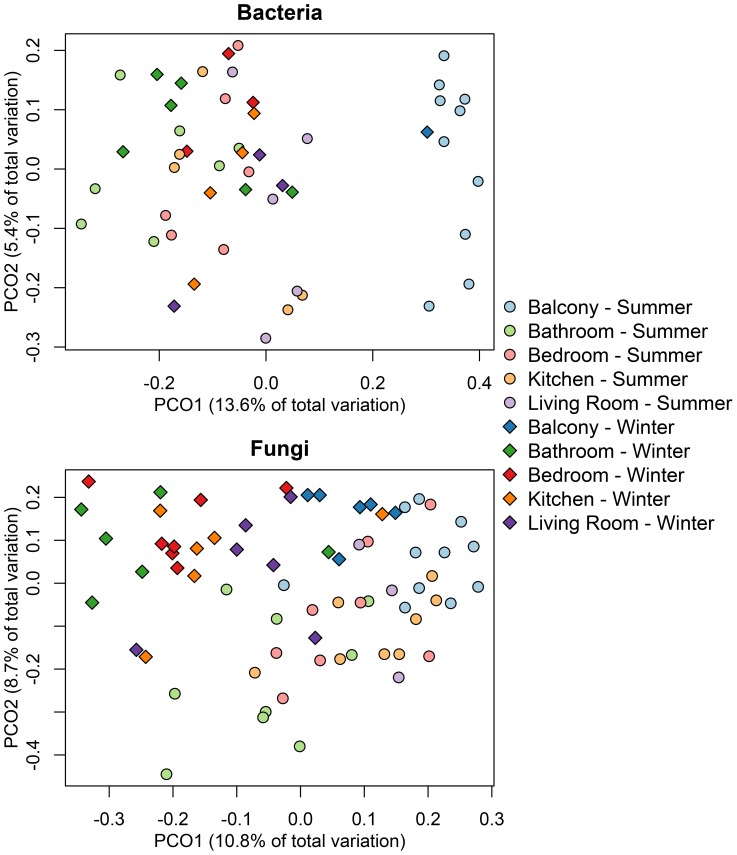
Visualization of differences in bacterial (A) and fungal (B) community composition based on binary Bray-Curtis index. Principal coordinates plot showing relationship among samples, where summer samples are circles, winter samples are squares, and room locations are color-coded.

Community composition clustered broadly by indoor and outdoor samples, whether based on Bray-Curtis taxonomic distance (Figure 2A) or Unifrac phylogentic distance (Figure S2). Based on composition as determined by binary Bray-Curtis distance of indoor samples, the single biggest factor predicting bacterial community composition was the unit (i.e. the building: ADONIS, df = 10, F. model = 1.46, R^2^ = 0.34, p = 0.01) followed only by the room type (ADONIS, df = 3, F. model = 1.24, R^2^ = 0.10, p = 0.02). No other factors − season, number of residents, age of building, sequencing run, use of humidifier, or presence of houseplant(s) − were found to be significant predictors, even after accounting for unit and/or room variation (ADONIS, p>0.05). Likewise, based on phylogenetic Unifrac distance, both unit (ADONIS, df = 10, F. model = 1.41, R^2^ = 0.33, p = 0.04) and room type (ADONIS, df = 3, F. model = 1.77, R^2^ = 0.13, p = 0.03) had a significant influence on bacterial community composition. For both metrics, unit and room type explained approximately 33% and 10%, respectively, of the variation in composition. Fungal community composition (Figure 2B) was explained by season (ADONIS, df = 1, F. model = 4.63, R^2^ = 0.07, p = 0.01) and unit (ADONIS, df = 10, F. model = 1.56, R^2^ = 0.26, p = 0.01). Room was a marginally significant predictor of indoor fungal community composition (ADONIS, df = 3, F. model = 1.26, R^2^ = 0.06, p = 0.08.

**Figure 3:**
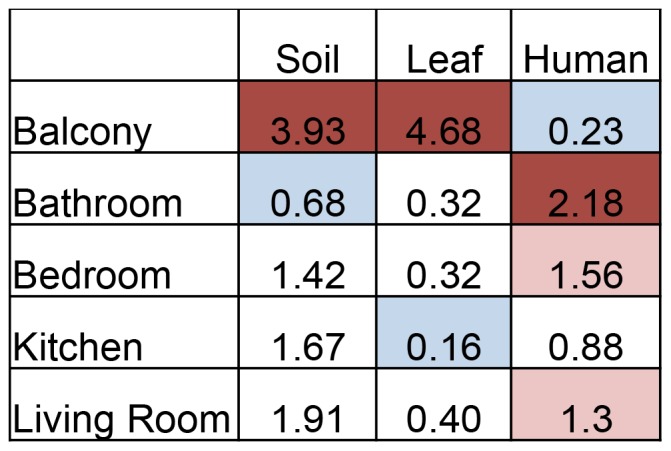
Heat map of the mean relative percentage of bacteria from a particular source (top row) at each sampling locations (left column). A red shade indicates a great influence of a particular source at a particular site, and a blue shad a less influence. Soil and leaf bacterial communities are a greater influence on balcony samples than indoor rooms. Conversely, human-associated bacteria are a greater influence on indoor samples than outdoor samples. Due to uncertainty in the source tracking approach, in which each taxon could not be assigned to a particular source (soil, leaf, human, or otherwise), this heatmap shows the different relative contributions of identified sources to different rooms, not different sources for a given room. In other words, the data should be read as columns, not rows.

**Figure 4:**
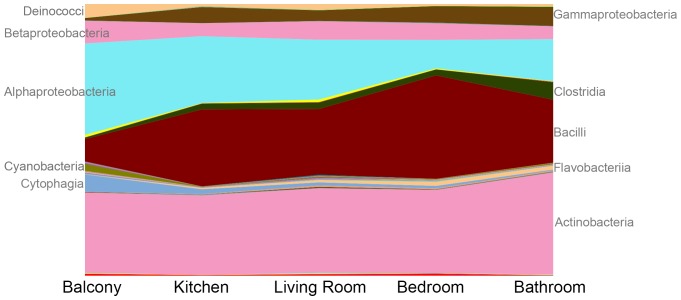
Taxonomic summary plots of bacterial orders across the different sampling locations. Movement from the left to the right of the graph follows typical steps from the doorway in the dwellings, where kitchens were closest to the front door and bathrooms were the most internal.

A source-tracking approach identified a total of 16 bacterial families associated with four different potential source populations (Table S3). Soil and leaf bacterial communities dominated the balcony samples, while human-derived bacteria were highly abundant in the indoor samples, particularly the bathroom, bedroom, and living room (Figure 3). The shift from outdoor-derived bacteria to human-associated species is also suggested in an area chart showing the composition of different bacterial classes across the different room types (Figure 4). In these residences, the entryway to the unit is adjacent to the kitchen and living room, while the bedroom and bathroom are situated down a hallway or up a flight of stairs. Taxa such as *Deinococci*, *Alphaproteobacteria*, *Cyanobacteria*, and *Cytophagia* are at their greatest relative abundance outdoors and decrease as foot traffic enters the indoor spaces, while *Gammaproteobacteria*, *Clostridia*, *Bacilli*, *Flavobacteria*, and *Actinobacteria* increase in abundance as you move to the more internal rooms of the dwelling. Within these broad taxonomic groups are shifts in relative abundance at finer taxonomic resolutions. For example, while the *Actinobacteria* appear to increase only slightly in the internal rooms, the outdoor Actinomycetes are comprised of the *Geodermatophilaceae* and *Nocardiaceae* familes, which are associated with stone and soil, and the indoors is dominated by the skin associated *Corynebacteriaceae, Propionibacteriaceae,* and *Streptomycetaceae*.

We also observed a weak but significant, positive relationship between differences in community composition and geographic distance; that is, a distance-decay effect could be detected over the small scale of hundreds of meters (Figure S3). The strength of the relationship increased for the summer indoor and outdoor samples (Figure S4) when taxa from the 12 human-associated bacterial families (Table S3) were removed from the community table. There was also a significant, positive relationship between bacterial and fungal community composition, such that those samples that had high bacterial composition distance also tended to have high fungal composition distance (42 common samples: r = 0.38, p<0.01; Figure S5).

### Bacterial Biomass

Patterns in biomass, as approximated by bacterial-primer amplification and therefore including of chloroplast amplicons, followed OTU richness where approximated biomass was higher outdoors than indoors (Mann-Whitney test, p<0.01) but not different among the different indoor room types (Kruskal-Wallis test, p>0.05).

## Discussion

The consideration of both bacteria and fungi from the same observational samples of indoor air has shown important differences between the sources for these microbes. Whereas indoor fungi reflect outdoor taxa with little contribution from the inhabitants, indoor bacteria are composed of outdoor taxa and taxa released from inhabitants. Bacteria in residences show more similarity to outdoor assemblages in the entryway and in rooms opening on the entryway, and a stronger signal of residents in bathrooms and bedrooms. Indoor and outdoor fungal assemblages show a decay in similarity as geographic distance increases and the same decay can be detected for bacteria, albeit more strongly when human associated taxa are excluded from the analysis and for summer months. The combined contribution of outdoor and indoor bacteria to indoor air is also shown by the only two residential features that explained significant variance in indoor bacterial assemblages: the unit, which is influenced by geographic distance; and the room type, which is structured by floor plan as well as likely differences in resident activity.

### Comparison with other Bacterial Indoor Microbiome Studies

In several ways, our results are generally concordant with other studies examining bacterial communities in indoor spaces. Overall microbial richness and biomass tends to be higher outdoors than indoors [6], [25]. The absolute observed richness in our samples, which were collected over a discrete period, is predictably less than those collected by swabbing similar residential surfaces for dust that has deposited over longer time frames [6], although some of these richness differences may also be due to analytical methods, including different sequencing depth and bioinformatic pipelines [12]. Like bacteria of different surfaces around the home [6], different rooms across residences harbor slightly different bacterial communities, indicating that different rooms demonstrate differential physical filtering from a common source, have different sources, and/or support different communities of endogenous growth. The measured house and resident characteristics, such as number of rooms or presence of houseplants, are either unimportant factors or replication numbers are too low to detect differences.

A strong contribution from people is consistently noted for studies on the indoor bacterial biome. Early work based on culturing bacteria note the importance of human-associated sources [26], including work from the late 1800s showing that overcrowding in schools and dust-raising activities that are more associated with boys than with girls can structure indoor bacterial communities [27]. It was also shown that in the Sistine Chapel bacterial concentrations correlative positively with the number of people [28]. Newer studies based on culture-free techniques also show that occupancy produces a distinct human signature [2], [4], [6], [7], [29], including one by Taubel et al. [29] which directly compared bacteria on skin swabs of residents and in their house dust. In the present study, the finding that the distance-decay effect increases when the recognized human-associated taxa are excluded suggests that at this level of resolution the human bacterial signature is a general one that dilutes a spatially structured background signature from outdoor sources.

The typical approach to sequencing with high-throughput technologies is to pool different barcoded-samples, each at a common amplicon concentration. Our previous work has shown however, that differential amplification can affect interpretation of compositional differences when concentrations in the original sample vary, such that high biomass samples are in effect sequenced to a less degree than low biomass samples [30]. This bias is probably operating here, to the effect that balcony samples are under-sequenced relative to the kitchen, living room, bedroom, and bathroom locations. Moreover, the amplification and subsequent exclusion of chloroplasts would also lead to an under-representation of bacteria in the outdoor samples, particularly in the winter. The richness of outdoor samples was then probably under-estimated (Figure 1A), and it is likely that a fuller sequencing depth would strengthen the pattern of increased taxa richness in outdoor samples.

### Comparison with other Fungal Indoor Microbiome Studies

Interestingly, traditional culture-based surveys of the built environment have focused on fungi [31] because of their health effects [32] while modern high-throughput surveys of bacteria in indoor locations are more common [3], [4], [6], [7]. The analysis of the same samples for both fungi and bacteria, as this study allows, is a useful design in that it allows a comparison of the processes that structure these two types of microorganisms. First, both bacteria and fungi exhibit higher richness and biomass outdoors relative to indoors [26]. Interestingly, both bacterial richness in aerosols and fungal richness on window sills tend to be higher for those units that report occasional humidifier use [33]. The mechanism leading to his correlation – such as whether *in situ* growth is greater or humidity affects bioaerosol viability, transport, or detection, or both – will require further work to elucidate. Second, both bacteria and fungi from these same samples showed a spatial structure in airborne communities. In both, building unit was the largest predictor of community similarity, and a distance-decay pattern is seen in the balcony (i.e., outdoor) samples as well as the indoor samples (Figure S3; Figure 2 in [10]). Thus the outdoor sources are spatially heterogeneous in both fungi and bacteria on relatively small spatial scales.

On the other hand, the strong human presence of bacterial taxa is unmatched with fungal taxa. While both show evidence for spatial heterogeneity, in contrast to bacteria no human-associated signal dampened the fungal distance-decay effect. Plus, while bacterial community composition shows little seasonality (see above), fungal taxa exhibit a prominent effect of collection timing, with richness being higher in the winter and with compositional differences almost completely distinct between the two seasons [10]. (These mild season differences, or even greater diversity in the winter in the case of fungi, is likely due to the particular climate of the study location, and different climates would be expected to show a different pattern; see, for example [34]). Human-associated fungi are only rarely (less than 6% of all sequences or clones) collected in airborne dust and vacuumed dust [1], [10], [11], [35]. In contrast, just one human-associated bacterial genus, *Corynebacterium,* represents 11% of our indoor sequences. Clearly, the input from humans as a source for indoor microbes is greater for bacteria than fungi.

## Conclusions

Seasonality and outdoor input appears much stronger for indoor bioaersols of fungi than bacteria, but this difference may be driven by the larger input of human-associated bacteria compared to fungi. For bacteria this signal from human “detritus” occurs on top of, and obscures, a local geographic patterning of bacterial communities that would otherwise show a stronger distance-decay effect similar to that seen in the fungi. We predict that if samples were sequenced to saturation and human-associated taxa could be definitely identified, fungi and bacteria would show a common biogeographical structure.

## Supporting Information

Figure S1 **Correlation between quanititative PCR and amplicon concentration after pyrosequencing PCR.** Concentration of amplified product was determined after uniform PCR conditions across the different types, as determined by Qubit and given as ug/ml. Biomass was determined by spore equivalents measured by the Real-time PCR. Correlation of the two is highly correlated (r = 0.78), and group summaries produce identical relative patterns.
(TIF)

Figure S2 **Visualization of differences in bacterial community composition based on weighted-Unifrac differences.**
(TIF)

Figure S3 **Correlations between geographic distance and community composition differences.** Values in left column are based on weighted Unifrac distances, and in right column on binary Bray-Curtis. Correlations were determined by mantel tests, and the mantel statistic (r) and significance are given for each calculation.
(TIF)

Figure S4 **Correlations between geographic distance and community composition differences with recognized human-associated taxa removed from the communities.** Values in left column are based on weighted Unifrac distances, and in right column on binary Bray-Curtis. Correlations were determined by mantel tests, and the mantel statistic (r) and significance are given for each calculation.
(TIF)

Figure S5 **Correlation between bacterial community distance and fungal community composition for those samples with both communities successfully sequenced.**
(TIF)

Table S1 **Information for each of the samples from the dwellings.**
(XLSX)

Table S2 **Bacterial OTUs in negative controls and excluded from other samples.**
(DOCX)

Table S3 **Bacterial families indicative of different sources, and their mean relative abundance across the different surface types. Indicator taxa follow those used in** [**6**] **and** [**9**] **.**
(DOCX)

Protocol S1 **Nucleic acid extraction and PCR-amplification details.**
(DOCX)
