# Supplementary figures and images for: Airborne Bacterial Communities in Residences: Similarities and Differences with Fungi
Source: PLoS One. 2014 Mar 6;9(3):e91283. doi: 10.1371/journal.pone.0091283 (PMC3946336; doi:10.1371/journal.pone.0091283)

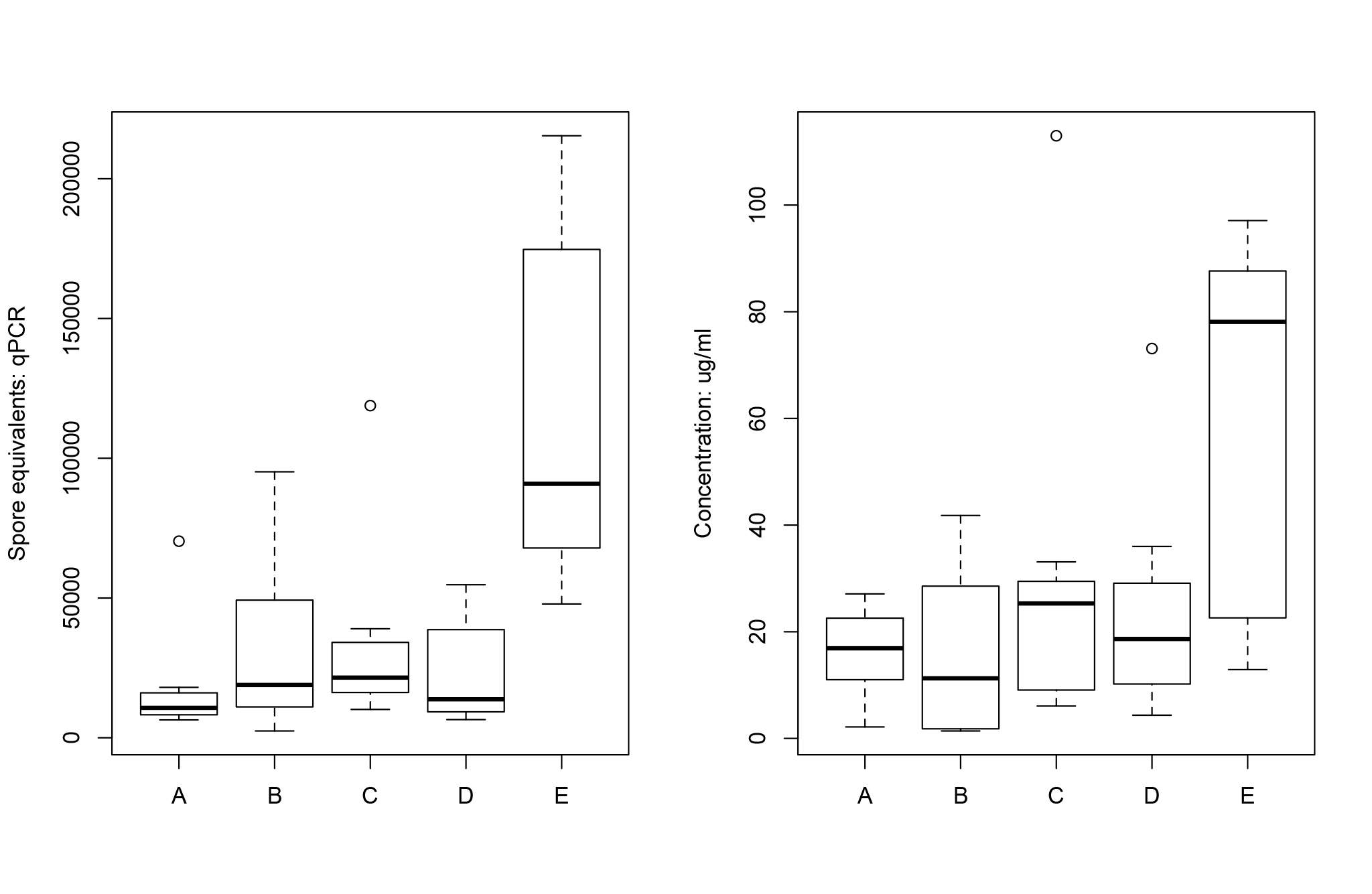

Supplement: Figure S1 — Correlation between quanititative PCR and amplicon concentration after pyrosequencing PCR. Concentration of amplified product was determined after uniform PCR conditions across the different types, as determined by Qubit and given as ug/ml. Biomass was determined by spore equivalents measured by the Real-time PCR. Correlation of the two is highly correlated (r = 0.78), and group summaries produce identical relative patterns. (TIF) [file pone.0091283.s001.tif]

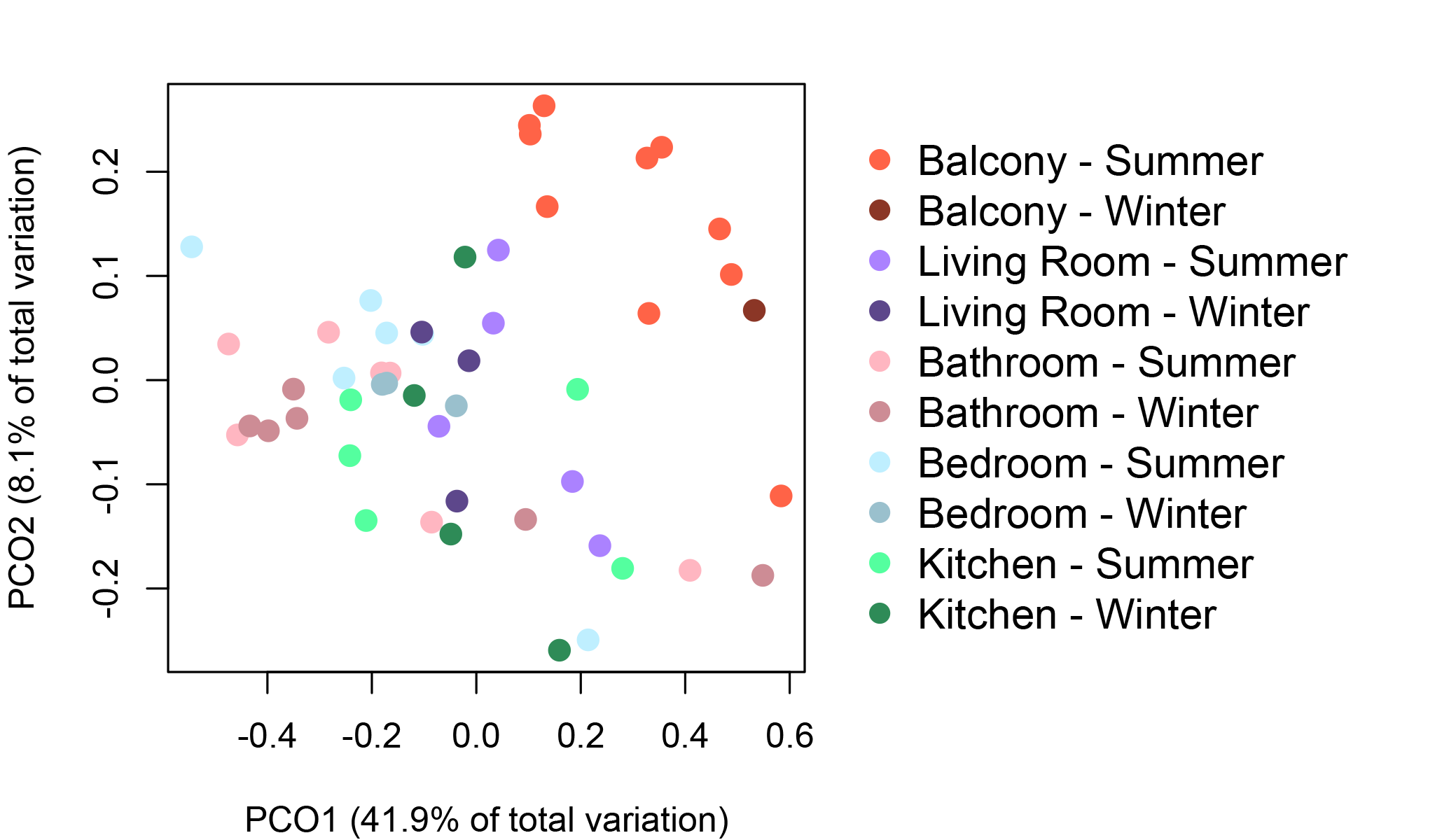

Supplement: Figure S2 — Visualization of differences in bacterial community composition based on weighted-Unifrac differences. (TIF) [file pone.0091283.s002.tif]

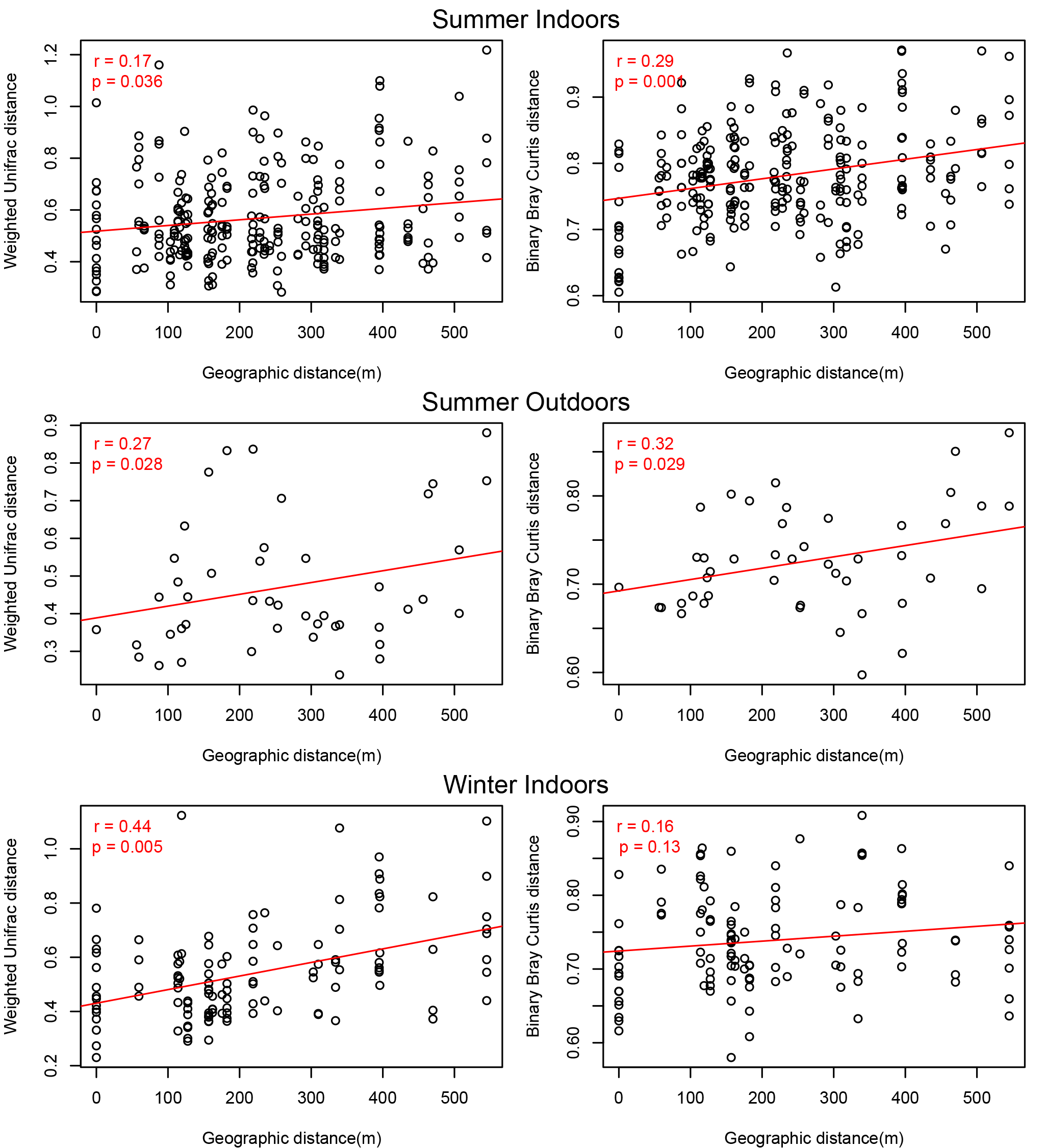

Supplement: Figure S3 — Correlations between geographic distance and community composition differences. Values in left column are based on weighted Unifrac distances, and in right column on binary Bray-Curtis. Correlations were determined by mantel tests, and the mantel statistic (r) and significance are given for each calculation. (TIF) [file pone.0091283.s003.tif]

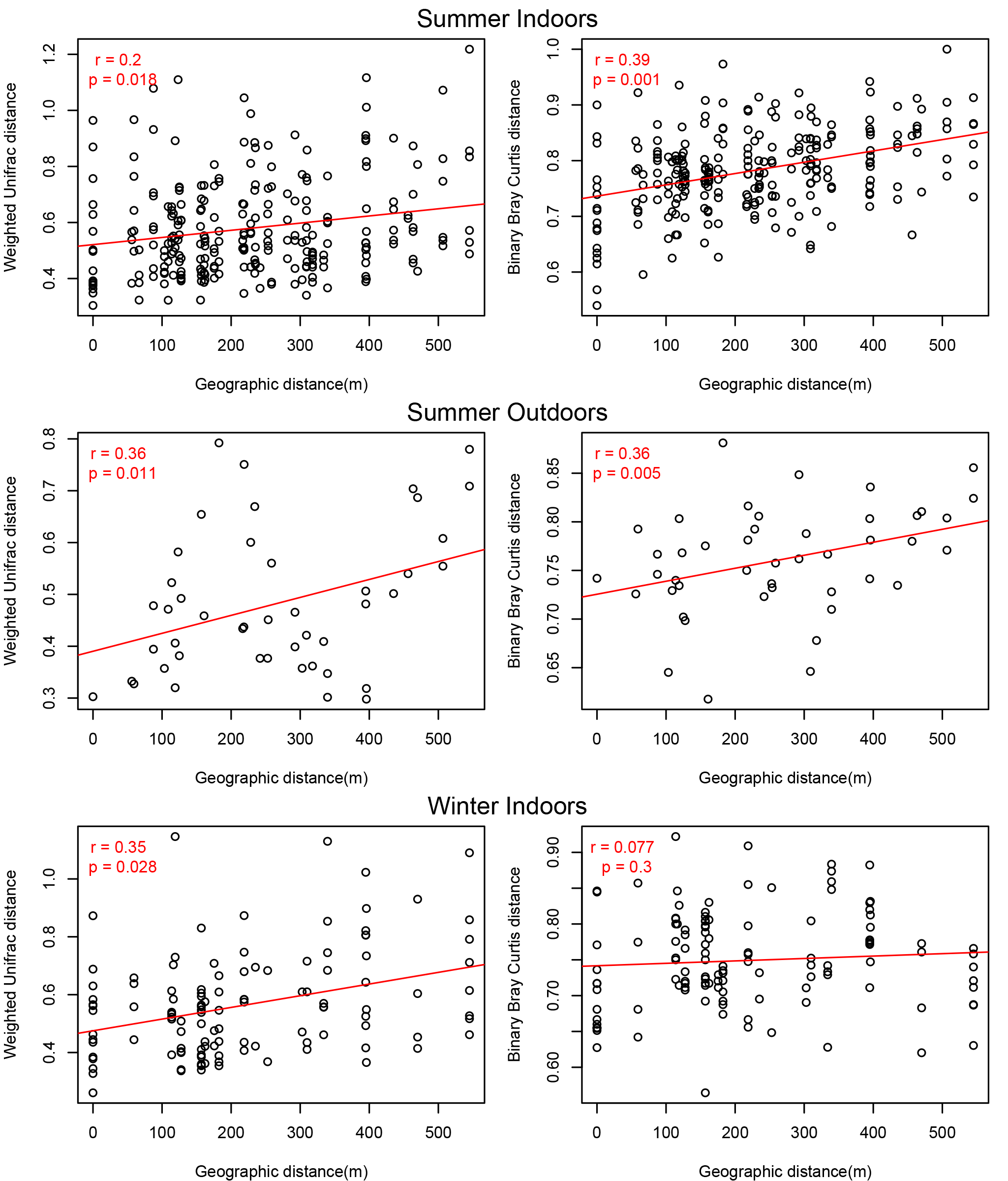

Supplement: Figure S4 — Correlations between geographic distance and community composition differences with recognized human-associated taxa removed from the communities. Values in left column are based on weighted Unifrac distances, and in right column on binary Bray-Curtis. Correlations were determined by mantel tests, and the mantel statistic (r) and significance are given for each calculation. (TIF) [file pone.0091283.s004.tif]

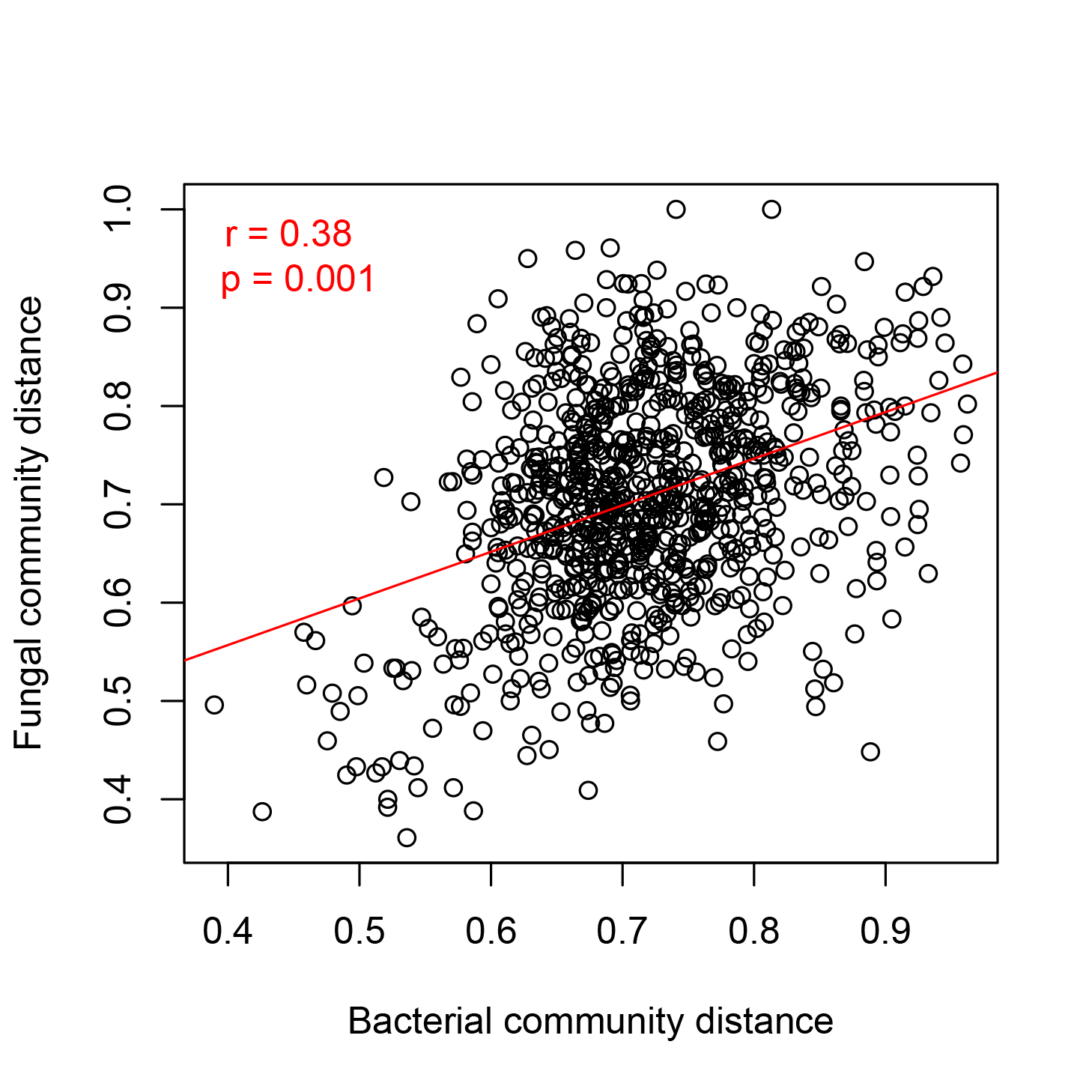

Supplement: Figure S5 — Correlation between bacterial community distance and fungal community composition for those samples with both communities successfully sequenced. (TIF) [file pone.0091283.s005.tif]
