# Supplementary material for: Airborne Bacterial Communities in Residences: Similarities and Differences with Fungi
Source: PLoS One. 2014 Mar 6;9(3):e91283. doi: 10.1371/journal.pone.0091283 (PMC3946336; doi:10.1371/journal.pone.0091283)
Supplement: Table S2 — Bacterial OTUs in negative controls and excluded from other samples. (DOCX) [file pone.0091283.s007.docx]

| **Taxonomy** | **Sequence** |
| --- | --- |
| Bacteria; [Thermi]; Deinococci; Thermales; Thermaceae; Thermus; | GGAGTGGGGCCCGTGTCTCAGTGCCCCTGTGGCCGGCCATCCTCTCAGACCGGCTACCCGTCGTCGCCTTGGTGGGCCAT  TACCCCACCAACTAGCTGATGGGACGCGGGCCCATCCGGAAGCGGGCAAAGCCCTTTGGACacacCCCAA |
| Bacteria; Proteobacteria; Alphaproteobacteria; Sphingomonadales; Sphingomonadaceae | GGAGTCTGGGCCGTGTCTCAGTCCCAGTGTGGCTGATCATCCTCTCAGACCAGCTATGGATCGTCGCCTTGGTAGGCTTT  TACCCCACCAACTAGCTAATCCAACGCGGGCTCATCCTTGGGCAATAAATCTTTGGTCTTGCGACATTAT |
| Bacteria; Proteobacteria; Gammaproteobacteria; Pseudomonadales; Moraxellaceae; Acinetobacter; rhizosphaerae | GGAGTCTGGGCCGTGTCTCAGTCCCAGTGTGGCGGATCATCCTCTCAGACCCGCTACAGATCGTCGCCTTGGTAGGCCTT  TACCCCACCAACTAGCTAATCCGACTTAGGCTCATCTATTAGCGCAAGGTCCGAAGATCCCCTGCTTTCT |
| Bacteria; Proteobacteria; Gammaproteobacteria; Xanthomonadales; Xanthomonadaceae | GGAGTCTGGACCGTGTCTCAGTTCCAGTGTGGCTGATCATCCTCTCAGACCAGCTACGGATCGTCGCCTTGGTGGGCCTT  TACCCCGCCAACTAGCTAATCCGACATCGGCTCATCTATCCGCGCAAGGCCCGAAGGTCCCCTGCTTTCA |
| Bacteria; Actinobacteria; Actinobacteria; Actinomycetales; Propionibacteriaceae; Propionibacterium; acnes | GGAGTCTGGGCCGTATCTCAGTCCCAATGTGGCCGGTCACCCTCTCAGGCCGGCTACCCGTCAAAGCCTTGGTAAGCCAC  TACCCCACCAACAAGCTGATAAGCCGCGAGTCCATCCCCAACCGCCGAAACTTTCCAACCcccACCATGC |
| Bacteria; Proteobacteria; Alphaproteobacteria; Rhizobiales; Bradyrhizobiaceae; Bosea; genosp. | GGAGTTTGGGCCGTGTCTCAGTCCCAATGTGGCTGATCATCCTCTCAGACCAGCTACTGATCGTCGCCTTGGTAGGCCAT  TACCCTACCAACTAGCTAATCAGACGCGGGCCGATCTTTCGGCGATAAATCTTTCCCCGTAAGGGCTTAT |
| Bacteria; Proteobacteria; Alphaproteobacteria; Rhizobiales; Methylobacteriaceae; Methylobacterium; | GGAGTCTGGGCCGTGTCTCAGTCCCAGTGTGGCTGATCATCCTCTCAGACCAGCTACTGATCGTCGCCTTGGTAGGCCGT  TACCCCACCAACTAGCTAATCAGACGCGGGCCGATCTTCCGGCAGTAAACCTTTCCCCAAAAGGGCGTAT |
| Bacteria; Proteobacteria; Betaproteobacteria; Burkholderiales; Alcaligenaceae; Achromobacter; | GGAGTCTGGGCCGTGTCTCAGTCCCAGTGTGGCTGGTCGTCCTCTCAAACCAGCTACGGATCGTCGCCTTGGTGAGCCGT  TACCCCACCAACTAGCTAATCCGATATCGGCCGCTCTAATAGTGCAAGGTCTTGCGATCCCCTGCTTTCC |
| Bacteria; Proteobacteria; Gammaproteobacteria; Xanthomonadales; Xanthomonadaceae; Stenotrophomonas; | GGAGTCTGGACCGTGTCTCAGTTCCAGTGTGGCTGATCATCCTCTCAGACCAGCTACGGATCGTCGCCTTGGTGGGCCTT  TACCCCGCCAACTAGCTAATCCGACATCGGCTCATTCAATCGCGCAAGGTCCGAAGATCCCCTGCTTTCA |
