# Supplementary material for: Airborne Bacterial Communities in Residences: Similarities and Differences with Fungi
Source: PLoS One. 2014 Mar 6;9(3):e91283. doi: 10.1371/journal.pone.0091283 (PMC3946336; doi:10.1371/journal.pone.0091283)
Supplement: Table S3 — Bacterial families indicative of different sources, and their mean relative abundance across the different surface types. Indicator taxa follow those used in [6] and [9] . (DOCX) [file pone.0091283.s008.docx]

**Table S3. Bacterial families indicative of different sources, and their mean relative abundance across the different surface types.** Indicator taxa follow those used in [[1](#_ENREF_1)] and [[2](#_ENREF_2)].

|  |  | Mean relative abundance (%) | | | | |
| --- | --- | --- | --- | --- | --- | --- |
| Source | Indicator taxon | Balcony | Bathroom | Bedroom | Kitchen | Living Room |
| Human oral | Pasteurellaceae | 0.00 | 0.28 | 0.32 | 0.40 | 0.55 |
| cavity | Fusobacteriaceae | 0.00 | 0.10 | 0.00 | 0.00 | 0.11 |
|  | Veillonellaceae | 0.00 | 0.28 | 0.20 | 0.16 | 0.15 |
|  | Neisseriaceae | 0.00 | 0.30 | 0.35 | 0.16 | 0.33 |
|  | Actinomycetaceae | 0.00 | 0.48 | 0.20 | 0.32 | 0.15 |
|  | Prevotellaceae | 0.00 | 0.24 | 0.17 | 0.08 | 0.07 |
| Human skin | Propionibacteriaceae | 1.66 | 5.83 | 2.48 | 1.50 | 1.75 |
|  | Staphylococcaceae | 0.98 | 2.70 | 2.54 | 1.58 | 1.72 |
|  | Corynebacteriaceae | 0.09 | 14.96 | 12.07 | 6.79 | 10.05 |
| Human stool | Lachnospiraceae | 0.03 | 0.72 | 0.23 | 0.24 | 0.44 |
|  | Bacteroidaceae | 0.00 | 0.02 | 0.06 | 0.04 | 0.07 |
|  | Ruminococcaceae | 0.00 | 0.20 | 0.06 | 0.04 | 0.22 |
| Leaf | Deinococcaceae | 4.68 | 0.32 | 0.32 | 0.16 | 0.40 |
| Soil | Hyphomicrobiaceae | 0.37 | 0.10 | 0.29 | 0.32 | 0.11 |
|  | Cytophagaceae | 6.32 | 0.34 | 0.89 | 1.78 | 1.13 |
|  | Microbacteriaceae | 5.11 | 1.60 | 3.09 | 2.91 | 4.50 |

1. Dunn RR, Fierer N, Henley JB, Leff JW, Menninger HL (2013) Home Life: Factors structuring the bacterial diversity found within and between homes. PLoS ONE 8: e64133.

2. Flores GE, Bates ST, Knights D, Lauber CL, Stombaugh J, et al. (2011) Microbial biogeography of public restroom surfaces. PLoS ONE 6: e28132.
