# Supplementary material for: Airborne Bacterial Communities in Residences: Similarities and Differences with Fungi
Source: PLoS One. 2014 Mar 6;9(3):e91283. doi: 10.1371/journal.pone.0091283 (PMC3946336; doi:10.1371/journal.pone.0091283)
Supplement: Protocol S1 — Nucleic acid extraction and PCR-amplification details. (DOCX) [file pone.0091283.s009.docx]

Airborne bacterial communities in residences: contrasting patterns to fungi

Protocol S1.

Nucleic Acid Extraction

Dust collection

1. Moisten a cotton-tipped applicator with water, and swab the petri dish surface for 5 seconds in each of north-south and east-west directions.
2. Use an ethanol and flame-sterilized scissor to cut cotton-tip directly into 2ml screwtop tube.

Nucleic acid isolation and lysis

1. Add Lysis matrix beads (Lysing Matrix E Tube – MP Biomedicals), 400 μL Miller phosphate buffer, 400 μL Miller SDS buffer, and 450 μL phenol: chloroform: isoamyl alcohol (25:24:1) to 2ml tube.
2. Bead beat tubes for 1 minute and max speed.
3. Centrifuge tubes at 10,000g for 5 minutes at 4°C.
4. Transfer approximately 560 μL of the aqueous supernatant to a new tube.
5. Add 560 μL chloroform and mix by inversion for 5 seconds.
6. Centrifuge tubes at 10,000g for 5 minutes at 4°C.
7. Transfer 480 μL to a new tube without disturbing chloroform layer.

DNA concentration

1. Add 2 volumes (960 μL) of MoBio Solution C4 to tube.
2. Mix by inverting 5 times and flicking gently (so as not to shear long DNA molecules).
3. Load Spin Filter with 750 μL aliquot and centrifuge at 10,000g for 30 seconds. Discard flow-through and repeat with remainder of mixture. Discard flow-through.
4. Add 400 μL MoBio Solution C5 to the filter and centrifuge at 10,000g for 30 seconds.
5. Discard flow-through and centrifuge again at 10,000g for 1 minute.
6. Carefully place spin filter in a new 1.5-ml tube. Avoid splashing any C5 at this step.
7. Add 50 μL warm MoBio Solution C6 to the center of the white filter membrane.
8. Centrifuge tubes at 10,000g for 30 seconds.
9. Repeat with another 50ul of warm C6 for a total of 100ul elute.

Miller phosphate buffer: 100 mM NaH_2_PO_4_, ph 8.0

Miller SDS buffer: 100 mM NaCl (from solid), 500 mM Tris, ph 8.0 (from 1M solution), 10% SDS wt./vol. (from 20% solution)

Sterilize

PCR Amplification:

Primers:

F: 5’ - B-adaptor (CCTATCCCCTGTGTGCCTTGGCAGTCTCAG) + 8f (AGAGTTTGATCCTGGCTCAG) -3’

R: 5′- A-adaptor (CCATCTCATCCCTGCGTGTCTCCGACTCAG + 8bp MID barcode + 357r (CTGCTGCCTYCCGTA) - 3’

Reagents:

|  | ul per rxn |
| --- | --- |
| 10X HotStarTaq PCR buffer | 2.5 |
| 10X 200uM dNTPs | 2.5 |
| F primer (50uM) untagged | 0.1 |
| HotStarTaq (5units/ul) | 0.25 |
| 100mg/ml BSA | 0.25 |
| H20 | 13.4 |
| Master Mix | 19 |
| R primer (5uM) tagged | 1 |
| 1:10 diluted DNA | 5 |

Thermocycle:

95° for 15s

30 cycles of:

95° for 30s

50° for 30s

72° for 30s

72° for 10m

10° hold
